# Mitochondrial function in neuronal cells depends on p97/VCP/Cdc48-mediated quality control

**DOI:** 10.3389/fncel.2015.00016

**Published:** 2015-02-02

**Authors:** Lei Fang, Charles Hemion, Ana C. Pinho Ferreira Bento, Claudia C. Bippes, Josef Flammer, Albert Neutzner

**Affiliations:** ^1^Department of Biomedicine, University of BaselBasel, Switzerland; ^2^Department of Ophthalmology, University of BaselBasel, Switzerland

**Keywords:** mitochondria, p97/VCP, neuronal cells, quality control

## Abstract

Maintaining mitochondrial function is essential for neuronal survival and offers protection against neurodegeneration. Ubiquitin-mediated, proteasome-dependent protein degradation in the form of outer mitochondrial membrane associated degradation (OMMAD) was shown to play roles in maintenance of mitochondria on the level of proteostasis, but also mitophagy and cell death. Recently, the AAA-ATPase p97/VCP/Cdc48 was recognized as part of OMMAD acting as retrotranslocase of ubiquitinated mitochondrial proteins for proteasomal degradation. Thus, p97 likely plays a major role in mitochondrial maintenance. Support for this notion comes from mitochondrial dysfunction associated with amyotrophic lateral sclerosis and hereditary inclusion body myopathy associated with Paget disease of bone and frontotemporal dementia (IBMPFD) caused by p97 mutation. Using SH-SY5Y cells stably expressing p97 or dominant-negative p97^QQ^ treated with mitochondrial toxins rotenone, 6-OHDA, or Aβ-peptide as model for neuronal cells suffering from mitochondrial dysfunction, we found mitochondrial fragmentation under normal and stress conditions was significantly increased upon inactivation of p97. Furthermore, inactivation of p97 resulted in loss of mitochondrial membrane potential and increased production of reactive oxygen species (ROS). Under additional stress conditions, loss of mitochondrial membrane potential and increased ROS production was even more pronounced. Loss of mitochondrial fidelity upon inactivation of p97 was likely due to disturbed maintenance of mitochondrial proteostasis as the employed treatments neither induced mitophagy nor cell death. This was supported by the accumulation of oxidatively-damaged proteins on mitochondria in response to p97 inactivation. Dysfunction of p97 under normal and stress conditions in neuron-like cells severely impacts mitochondrial function, thus supporting for the first time a role for p97 as a major component of mitochondrial proteostasis.

## Introduction

Failing mitochondrial maintenance is at the heart of neurodegeneration and associated neuronal death (Karbowski and Neutzner, 2012; Kornmann, 2014). To prevent the untimely death of neuronal cells, mitochondria are kept healthy and in shape through the complex interplay of various molecular mechanisms aimed at repairing mitochondrial damage on the molecular level (Anand et al., 2013) or at removing damaged mitochondrial subunits from the cell (Youle and Narendra, 2011). A key component of mitochondrial maintenance is quality control of damaged, dysfunctional proteins through protein degradation (Neutzner et al., 2008; Escobar-Henriques and Langer, 2014). Owed to the complex architecture and the endosymbiotic nature of mitochondria, several protein degradation mechanisms are in place to maintain proteostasis in the various mitochondrial compartments (Tatsuta and Langer, 2008). Recently, we and others described roles for the ubiquitin-proteasome system in maintaining mitochondrial function and proteostatis. E3 enzymes, namely MARCH5/MITOL (Karbowski et al., 2007; Nagashima et al., 2014), MAPL/MULAN (Braschi et al., 2009), IBRDC2 (Benard et al., 2010), RNF185 (Tang et al., 2011), and Parkin (Narendra et al., 2008) were found to localize to the mitochondrial outer membrane. Furthermore, ubiquitin-dependent protein degradation was shown to modulate mitochondrial morphology (Neutzner and Youle, 2005; Karbowski et al., 2007; Cohen et al., 2008; Leboucher et al., 2012) and impact mitophagy (Narendra et al., 2012). In addition, quality control of mitochondria-localized poly-Q (Sugiura et al., 2011), amyotrophic lateral sclerosis associated mSOD1 as well as S-nitrosylated proteins (Benischke et al., 2014) is performed by the ubiquitin-proteasome system. Analogous to the endoplasmic reticulum (ER) which is quality controlled by ER associated degradation or ERAD (Ruggiano et al., 2014), mitochondria might be considered to be under control of outer mitochondrial membrane associated degradation (OMMAD, Neutzner et al., 2007).

Quality control of proteins localized to a membrane-bound organelle by the cytosolic ubiquitin-proteasome system must involve the protein extraction from the organelle and retrotranslocation into the cytosol for degradation. The AAA-ATPase valosin containing protein VCP/p97/Cdc48 is the central component of this retrotranslocation machinery necessary for proteasomal degradation of organellar proteins (Meyer et al., 2012). Interestingly, p97 fulfils this function for ERAD (Wolf and Stolz, 2012) and OMMAD (Xu et al., 2011) alike. As such, p97 is an integral part of proteasomal quality control of ER-localized as well as mitochondrial proteins. While the role of p97 in maintaining ER proteostasis is extensively studied, the connection between p97 and mitochondrial health is less clear. However, p97 dysfunction was recently linked to some forms of amyotrophic lateral sclerosis and hereditary inclusion body myopathy associated with Paget disease of bone and frontotemporal dementia (IBMPFD) and a connection to failed mitochondrial quality control was suspected (Yamanaka et al., 2012).

To further define the role of p97 in mitochondrial maintenance especially in neuronal-like cells, we studied the influence of p97 inactivation on mitochondrial health and function in SH-SY5Y cells in comparison to known neurotoxic mitochondrial insults. We found that inactivation of p97 negatively impacts mitochondrial function in terms of membrane potential, reactive oxygen production, morphological changes, and accumulation of oxidized proteins comparably to treatment with the electron transport chain inhibitor rotenone, the neurotoxin 6-hydroxydopamine as well as Alzheimer's disease related Aβ peptide. These findings support an important function for p97 in maintaining neuronal health through mitochondrial protein quality control and further strengthen the link between mitochondrial dysfunction and premature neuronal death.

## Results

### Mitochondrial fragmentation as result of inactivation of p97

**Figure 1:**
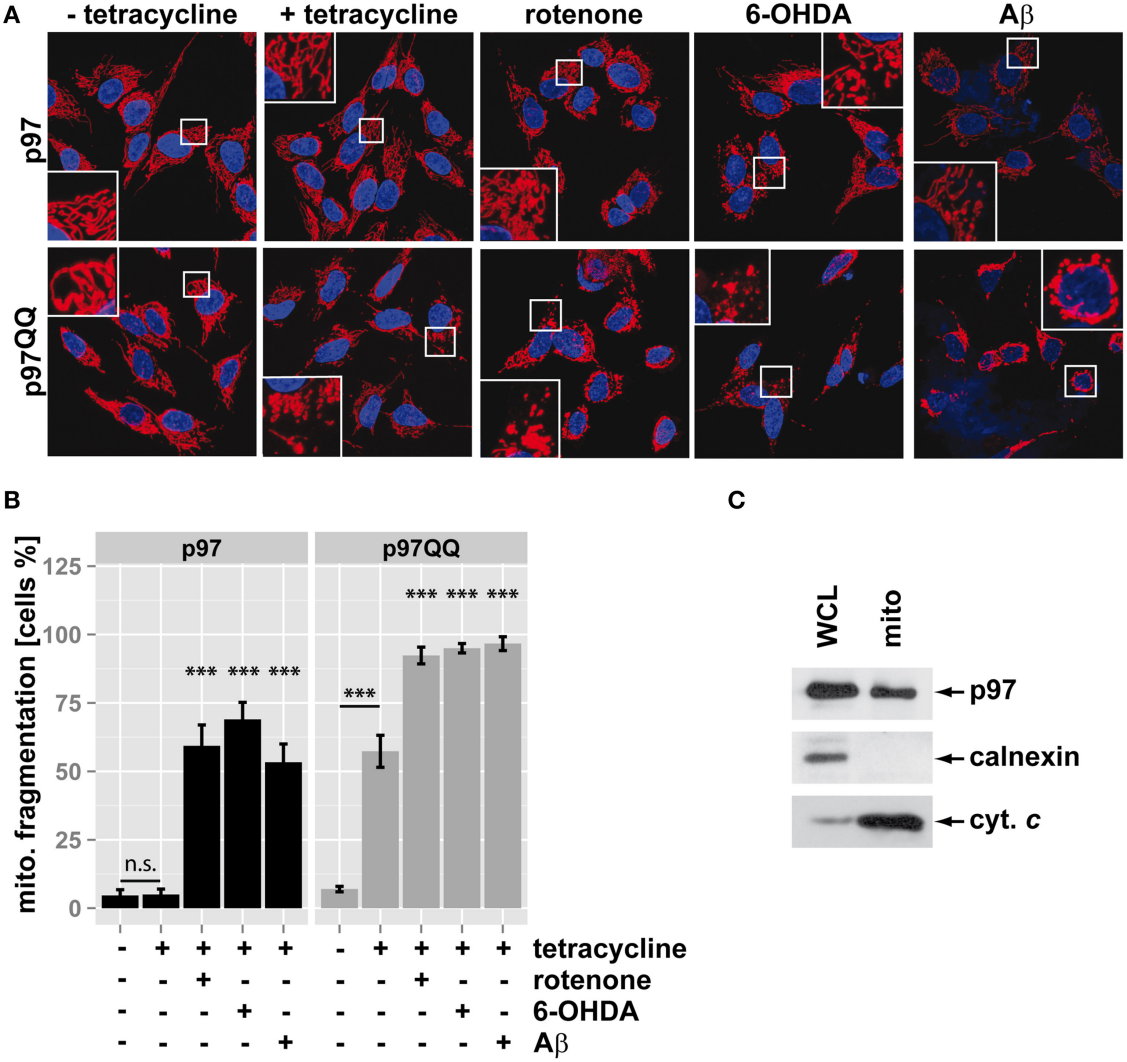
**Inactivation of p97 increased mitochondrial fragmentation under stress**. **(A)** SH-SY5Y cells stably expressing p97 or dominant-negative p97QQ under control of the Tet-On promoter were induced with tetracycline for 16 h or left uninduced and treated with 5 μM rotenone, 75 μM 6 OHDA, or 50 μM Aβ for 6 h. Cells were fixed and stained using anti-cytochrome c antibody and Alexa546-conjugated secondary antibody. Shown are representative pictures from three independent experiments. **(B)** Fragmentation of the mitochondrial network in cells from **(A)** was quantified by visual examination. Shown is the average of three independent experiments. Statistical analysis was performed using pair-wise *t*-tests with *p*-value adjustment according to Holm. Statistical significance is marked with n.s. for not significant, ^***^p < 0.001. **(C)** The presence of p97 on mitochondria was analyzed by western blotting using anti-p97 antibodies following purification of mitochondria using anti-TOMM22 magnetic beads. The purity of mitochondrial fractions was assessed by detecting the ER marker calnexin and the mitochondrial marker cytochrome c (cyt. c). Shown is one representative out of three independent experiments.

Mitochondria fragment in response to mitochondrial insults such as the complex I inhibitor rotenone, the neurotoxic compound 6-hydroxydopamine (6-OHDA) or the Alzheimer's-related peptide Aβ. To assess whether p97 is involved in mitochondrial maintenance and stress protection in neuron-like cells, p97 function was blocked by overexpression of p97^QQ^, a dominant-negative version of p97, under conditions of mitochondrial insult. In order to induce low level mitochondrial insult, toxin concentration and time of insult was selected to minimize impact on cellular viability. To this end, SH-SY5Y neuroblastoma cells stably expressing p97 or dominant-negative p97^QQ^ under control of a tetracycline-inducible promoter were treated with tetracycline in the presence of rotenone, 6-OHDA, or Aβ. As shown in Figure 1, expression of p97^QQ^ caused mitochondrial fragmentation in 57.3 ± 5.9% compared to 5 ± 2% in control cells expressing p97 and 7 ± 1% in uninduced p97^QQ^ cells. Treatment of p97-expressing cells with the mitochondrial toxins rotenone, 6-OHDA or Aβ resulted in mitochondrial fragmentation in 59.3 ± 7.6%, 69 ± 6.2%, and 53.3 ± 6.7%, respectively. Interestingly, expression of p97^QQ^ in the presence of mitochondrial toxins had a strong additive effect and caused mitochondrial fragmentation in 92.3 ± 3.1% (rotenone), 95 ± 1.7% (6-OHDA), and 96.7 ± 2.5% (Aβ) of SH-SY5Y cells. These data are consistent with a role for p97 in mitochondrial maintenance during normal as well as under mitochondrial stress conditions. To further explore this function of p97 on mitochondria in neuron-like cells, highly purified mitochondria were analyzed by western blotting for the presence of p97. As shown in Figure 1C, p97 was found in mitochondrial fractions from SH-SY5Y cells enriched in the mitochondrial marker cytochrome c, but devoid of the ER-marker calnexin. These data are consistent with p97 present on mitochondria and confirm previous observations of mitochondrial p97 (Xu et al., 2011).

### Inactivation of p97 negatively impacts mitochondrial membrane potential and increases production of reactive oxygen species

**Figure 2:**
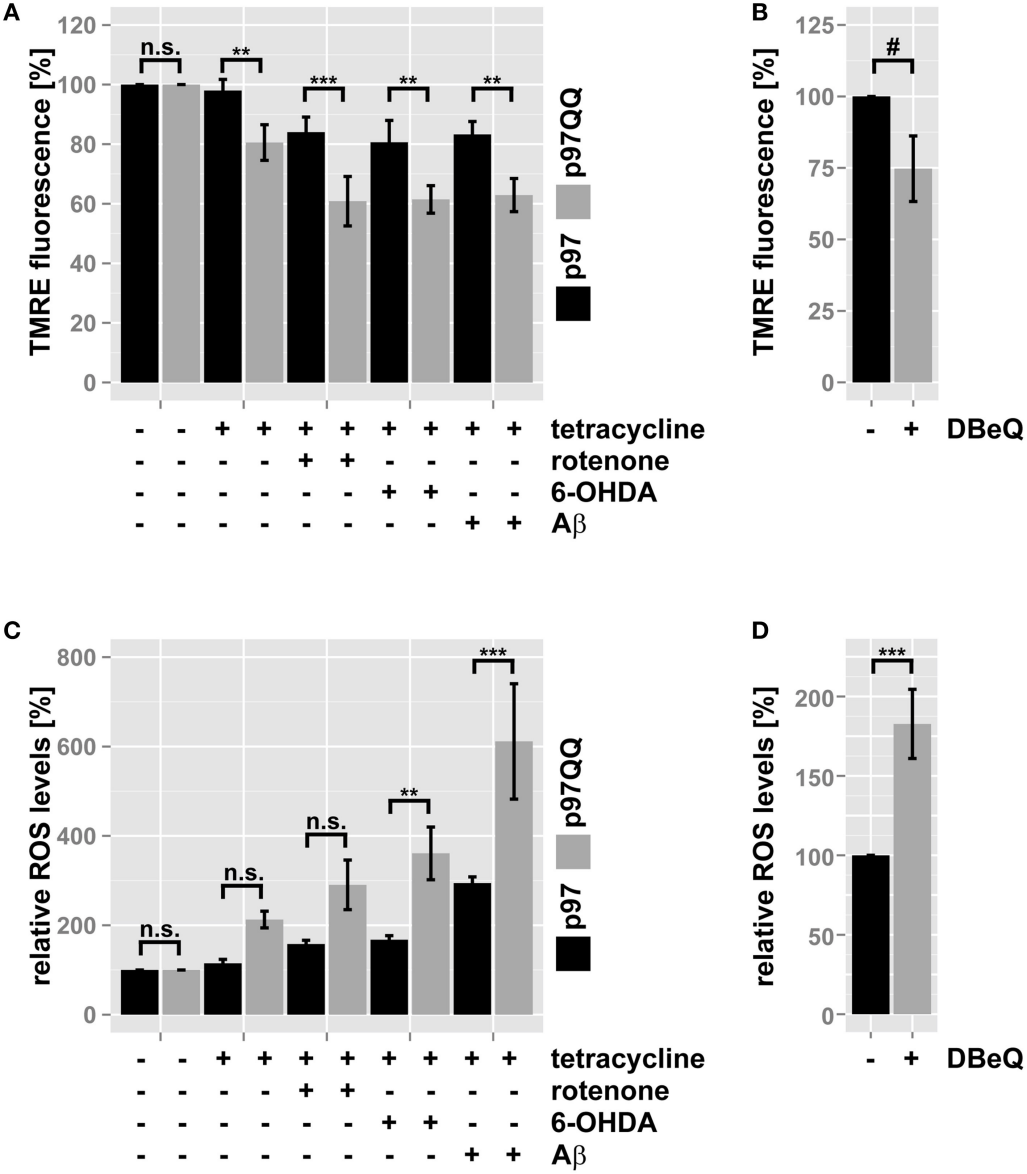
**Inactivation of p97 impairs mitochondrial function during neurotoxic stress**. **(A)** SH-SY5Y cells stably expressing p97 or dominant-negative p97QQ under control of the Tet-On promoter were induced with tetracycline for 2 h or left uninduced and treated with 5 μM rotenone, 75 μM 6 OHDA, or 50 μM Aβ for an additional 6 h. Cells were stained with the mitochondrial membrane sensitive dye TMRE and analyzed by flow cytometry. **(B)** SH-SY5Y cells were treated with the p97 inhibitor DBeQ and mitochondrial membrane potential was measured by flow cytometric analysis of TMRE fluorescence. **(C)** Cells treated as in **(A)** were stained with the ROS-sensitive dye MitoSox and mitochondrial ROS generation was measured using flow cytometry. **(D)** SH-SY5Y cells were treated with the p97 inhibitor DBeQ and mitochondrial ROS generation was measured by flow cytometric analysis of MitoSox fluorescence. Statistical analysis was performed using pair-wise *t*-tests with Holm *p*-value adjustment for **(A,C)**, and a general linear model (SPSS) for **(B,D)**. Statistical significance is marked with n.s. for not significant, ^#^*p* < 0.1, ^**^p < 0.01, ^***^p < 0.001.

Mitochondrial membrane potential is a measure for mitochondrial health with a drop in membrane potential being a sign of mitochondrial dysfunction. Mitochondrial membrane potential was measured in neuron-like cells under conditions of mitochondrial insult during p97 inactivation to further assess the role of p97 in maintaining mitochondrial health. To this end, SH-SY5Y cells stably expressing p97 or p97^QQ^ were treated with rotenone, 6-OHDA, or Aβ or left untreated as control and mitochondrial membrane potential was measured using the membrane-potential sensitive dye tetramethylrhodamine ethyl ester (TMRE). As shown in Figure 2A, ectopic expression of p97 did not impact mitochondrial membrane potential compared to control, while the potential was diminished to 80.6 ± 6.0% of control by expression of p97^QQ^. Furthermore, treatment with mitochondrial toxins in addition to p97^QQ^ expression caused a further reduction of mitochondrial membrane potential to 60.9 ± 8.3% (rotenone), 61.5 ± 4.6% (6-OHDA), and 62.9 ± 5.6% (Aβ). Pharmacological inhibition of p97 was employed to confirm the observed impact of p97 inactivation on mitochondrial membrane potential. To this end, SH-SY5Y cells were treated with the specific p97 inhibitor DBeQ or vehicle (DMSO) and flow cytometric analysis of TMRE fluorescence was performed. As shown in Figure 2B, DBeQ-dependent inhibition of p97 caused a reduction of TMRE fluorescence to 74.7 ± 11.5 of control cells (*p* = 0.072) comparable to the drop of mitochondrial membrane potential following expression of p97^QQ^.

In addition to mitochondrial depolarization, increased production of reactive oxygen species (ROS) is a hallmark of failing mitochondrial maintenance and subsequent dysfunction. To further analyse the role of p97 in mitochondrial maintenance in neuron-like cells cells, ROS production was measured under mitochondrial stress conditions in the presence or absence of p97 function. Again, SH-SY5Y cells stably containing tetracycline-inducible p97 or p97^QQ^ were induced with tetracycline, treated with rotenone, 6-OHDA, or Aβ or left untreated as control, and mitochondrial ROS levels were analyzed by flow cytometry using the ROS-sensitive dye MitoSox. While ectopic expression of p97 had only a minor influence on ROS levels (114.9 ± 8.7%) compared to uninduced control cells (Figure 2C), expression of p97^QQ^ alone caused ROS levels to increase to 212.7 ± 18.7%. Additional treatment of p97 expressing cells with mitochondrial toxins further increased ROS levels to 158 ± 8.3% (rotenone), 167.7 ± 9% (6-OHDA), and 294.3 ± 14.1% (Aβ). In response to p97 inactivation under mitochondrial stress conditions ROS levels further increased to 290.4 ± 55.6% (rotenone), 360.9 ± 58.9% (6-OHDA), and 611.5 ± 129.1% (Aβ). As above, pharmacological inhibition was used to confirm the impact of p97 on mitochondrial ROS production (Figure 2D). Treatment with the p97 inhibitor DBeQ caused a highly significant 1.8 ± 0.2 fold increase in ROS production compared to vehicle treated control cells again confirming the observed elevated ROS levels following expression of p97^QQ^. The observed increase of ROS production following p97 inactivation and the significant additive effect on ROS production of inactive p97 during mitochondrial stress strongly support a crucial role for p97-dependent mitochondrial maintenance under normal as well as stress conditions.

### Inactivation of p97 impairs mitochondrial maintenance below the mitophagic threshold

**Figure 3:**
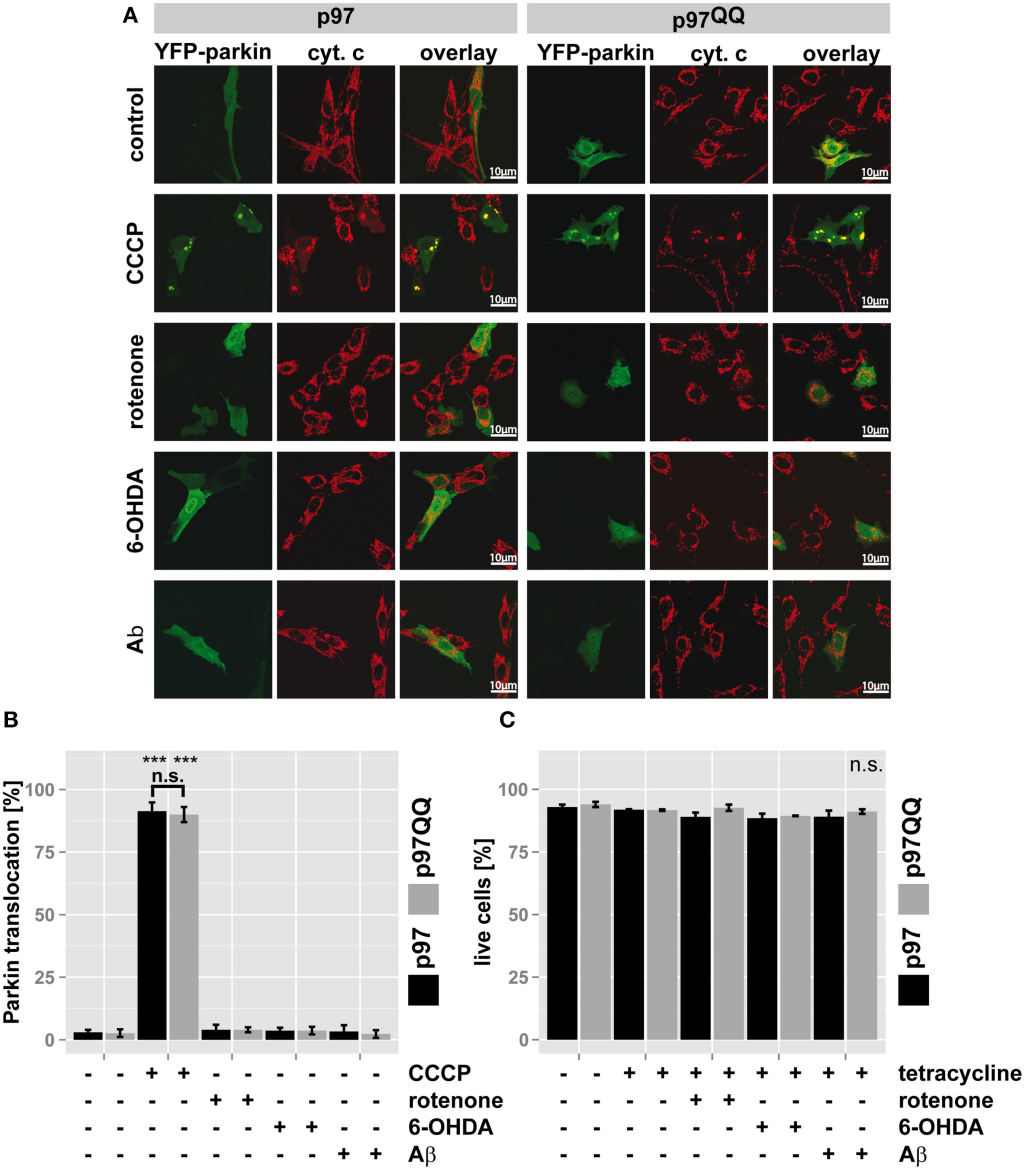
**p97 is involved in mitochondrial maintenance at damage levels below the thresholds for mitophagy and cell death**. **(A)** SH-SY5Y cells stably expressing p97 or dominant-negative p97QQ under control of the Tet-On promoter were transfected with an expression construct for YFP-tagged Parkin, induced with tetracycline for 2 h or left uninduced and treated with 5 μM rotenone, 75 μM 6 OHDA, or 50 μM Aβ for an additional 6 h. Cells were fixed, stained for the mitochondrial marker cytochrome c (cyt. c) and Parkin translocation from the cytosol to mitochondria was visually analyzed using fluorescence microscopy. Shown are representative images of three independent experiments. **(B)** Shown is a quantification of Parkin translocation from the cytosol to mitochondria from **(A)**. **(C)** Cells treated as in **(A)** were stained with the cell impermeable dye 4′,6-diamidino-2-phenylindole (DAPI) and the percentage of dead cells was determined by flow cytometry. Statistical analysis was performed using pair-wise *t*-tests with *p*-value adjustment according to Holm. Statistical significance is marked with ^***^p < 0.001. In **(C)** no statistical significance was observed.

Recently, p97 was implicated in the execution of mitophagy (Tanaka et al., 2010). To assess whether the observed mitochondrial dysfunction under the employed stress conditions and p97 inactivation are linked to blocked mitophagy or might be attributed to other roles of p97, Parkin translocation to mitochondria (Narendra et al., 2008) as a marker for mitophagic induction was measured. To this end, SH-SY5Y cells stably expressing p97 or p97^QQ^ were transfected with an expression construct for YFP-tagged Parkin, induced with tetracycline and exposed to mitochondrial insults by treating with rotenone, 6-OHDA, or Aβ. Treatment with the protonophore (3-Chlorophenyl)hydrazonomalononitrile (CCCP) capable of complete mitochondrial depolarization and subsequent Parkin translocation served as control. As shown in Figures 3A,B, while treatment with CCCP induced Parkin translocation in almost all cells, neither expression of p97 or p97^QQ^, nor neurotoxin treatment at the used concentrations caused significant (<4%) translocation of Parkin to mitochondria. Consistent with this finding, the employed concentrations of rotenone, 6-OHDA, and Aβ also did not lead to cell death as measured by flow cytometry (Figure 3C), neither in the presence of ectopic p97 nor following expression of p97^QQ^. Thus, while the employed treatments with neurotoxic substances degraded mitochondrial function, the threshold for mitophagic induction as well as cell death was not reached. Therefore, the observed role for p97 in mitochondrial maintenance under the stress conditions employed by us is also likely on the protein degradation level rather than on the mitophagic or cell death level.

### p97 is involved in clearing oxidatively-damaged proteins from mitochondria

**Figure 4:**
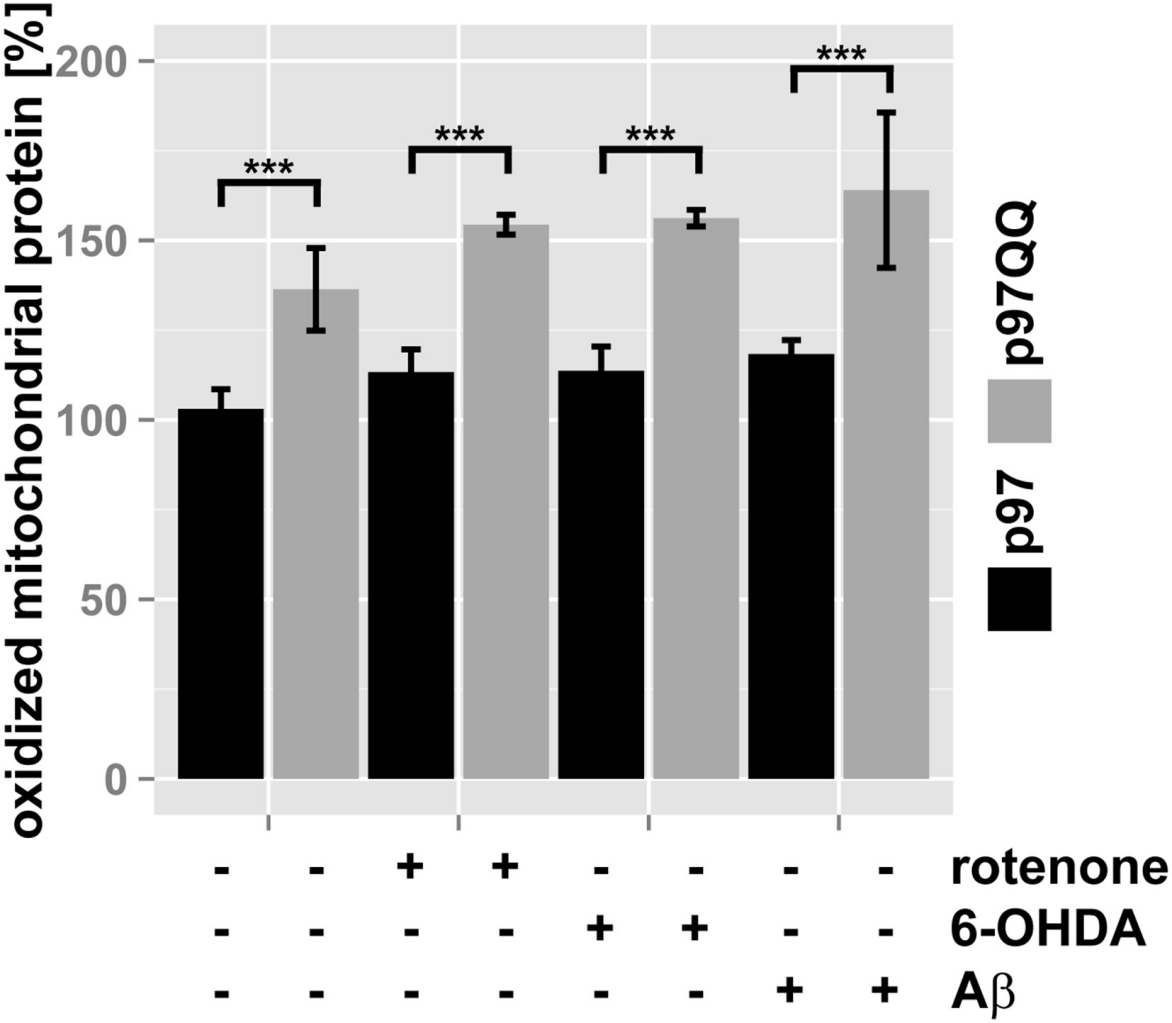
**Clearance of oxidatively-damaged mitochondrial proteins is impaired following inactivation of p97**. SH-SY5Y cells stably expressing p97 or dominant-negative p97QQ under control of the Tet-On promoter were induced with tetracycline for 2 h or left uninduced and treated with 5 μM rotenone, 75 μM 6 OHDA, or 50 μM Aβ for an additional 6 h. Mitochondria were isolated using anti-TOMM22 magnetic beads resulting in highly purified mitochondria. Protein carbonylation as measure for oxidative damage was determined by infrared laser-based quantitative western blotting following derivatization with 2,4-Dinitrophenylhydrazine (DNPH) and detection using anti-DNP antibodies. Shown is the average of three independent experiments. Please see Supplementary Figure S1 for representative western blot images. Statistical analysis was performed using pair-wise *t*-tests with *p*-value adjustment according to Holm. Comparisons shown are tetracycline-induced, untreated cells vs. tetracycline-induced, treated cells. Statistical significance is marked with n.s. for not significant, ^***^p < 0.001.

To further support this notion, the ubiquitin- and p97-mediated proteasome-dependent turnover of oxidized mitochondrial proteins under mitochondrial stress conditions was measured. In SH-SY5Y cells ectopically expressing p97, treatment with neither rotenone, nor 6-OHDA or Aβ significantly increased levels of oxidized proteins compared to untreated control cells (Figure 4, for representative western blots see Supplementary Figure S1). Interestingly, in p97^QQ^ expressing cells, levels of oxidized mitochondrial proteins were significantly increased (*p* < 0.001) compared to p97 expressing cells confirming a function for p97 in the removal of oxidatively-damaged mitochondrial proteins. Furthermore, levels of oxidized proteins significantly increased in p97^QQ^ expressing cells under mitochondrial stress conditions due to rotenone, 6-OHDA, and Aβ treatment compared to p97^QQ^ control cells. Again, these data are consistent with the involvement of p97 in the degradation of damaged proteins to maintain mitochondrial function in neuron-like cells under mitochondrial stress conditions below the threshold of mitophagic induction or cell death.

## Discussion

Mechanisms of mitochondrial maintenance act on the cellular level by removing complete dysfunctional mitochondrial networks through programmed cell death. On the organellar level they act through mitophagic degradation of dysfunctional mitochondrial subunits and finally on the molecular level by repairing and/or removing damaged mitochondrial components such as DNA, lipids or proteins (Tatsuta and Langer, 2008). While programmed cell death is an effective means to rid the body of damaged and, due to their excessive ROS production, potentially harmful mitochondrial networks, in post-mitotic neurons excessive apoptotic cell death is equivalent to neurodegeneration (Radi et al., 2014). Thus, mitophagy and degradation of damaged proteins are likely the first lines of defense against mitochondrial dysfunction in neurons as neuronal death is avoided. Which mechanism, mitophagy or protein degradation is more prevalent in neurons under normal conditions remains unclear. However, it is conceivable that the removal of individual components such as damaged proteins from otherwise functional mitochondria might be preferable to the destruction of whole mitochondrial subunits. Especially under conditions of everyday stress, with slowly accumulating, low overall mitochondrial damage, keeping mitochondrial damage below the mitophagic threshold through the constant removal of damaged proteins is likely to prevent neuronal damage.

Using known mitochondrial toxins such as rotenone, 6-OHDA, and Aβ at concentrations and treatment times below the threshold of cell death and mitophagic induction, we intended to model the above mentioned low level stress and evaluate the importance of p97 and associated protein degradation as well as its influence on mitochondrial fidelity in neuronal cells. Interestingly, inactivation of p97 alone—without additional exogenous stress–for as little as 8 h negatively influenced mitochondrial morphology, membrane potential and ROS production. Although p97 has many cellular functions and pleiotropic effects of p97 inactivation have to be taken into account, these observations are consistent with a direct role for p97 in mitochondrial maintenance under normal conditions in the absence of external mitochondrial stress. This notion is further supported by the accumulation of oxidatively-damaged mitochondrial proteins following p97 inactivation. Even under normal conditions without exogenous mitochondrial stress, a considerable amount of oxidized protein is present in mitochondria and significantly accumulates as consequence of a short period of p97 inactivation. Thus, in the absence of any detectable mitophagic activity, continuous turnover of damaged mitochondrial proteins occurs in a p97-dependent manner. As p97 is an ubiquitin-dependent chaperone, this is consistent with constantly ongoing mitochondrial maintenance in neuronal cells through the ubiquitin-proteasome system under normal, unstressed conditions. Consistent with this finding, we also found p97 to be present in mitochondrial fractions under normal conditions, and recently we also connected p97 and oxidatively-damaged proteins to OMMAD, the ubiquitin-dependent, proteasome-mediated degradation mitochondrial proteins (Hemion et al., 2014). Further support to this notion is lent by the effect of p97 inactivation under exogenous stress conditions below the mitophagic threshold. Concurrent stress and p97 inactivation had additive effects on mitochondrial fragmentation, membrane potential, ROS production as well as the accumulation of oxidatively-damaged mitochondrial proteins. These observations again support a function for p97 in dealing with mitochondrial damage to keep dysfunction of mitochondria below the threshold above which mitophagic degradation or even cell death occurs. Thus, we propose that constant repair of mitochondrial damage by the ubiquitin-proteasome system in a p97-dependent manner even under conditions of no or low exogenous mitochondrial stress is critically important for maintaining mitochondrial function under normal conditions. Mitophagic clearance on the other hand might be responsible for maintaining mitochondrial function in response to more drastic insults likely not encountered under normal conditions. Taken together, p97-mediated mitochondrial proteostasis is likely an important mechanism to prevent mitochondrial dysfunction as result of slowly accruing mitochondrial damage under normal conditions and to keep associated neurodegenerative processes at bay.

## Materials and methods

### DNA constructs

To generate a vector for the one-step generation of stable, tetracycline-inducible human cell lines, the CMV promoter in the AAVS1 donor cloning vector DC-DON-SH01 (Genome-TALER™ human AAVS1 safe harbor gene knock-in kit, GeneCopoeia) was replaced by the hybrid CMV/Tet-On promoter originating from pcDNA5/FRT/TO (Invitrogen) with *Mlu*I/*Pme*I. Afterwards, a stuffer sequence with 5′ *Pme*I site followed by *Eco*RV site and 3′ *Bst*BI site was inserted using *Pme*I, *Bst*BI to generate pAN2066. To enable tetracycline regulation of the construct, the *GFP* coding region in pAN2066 was replaced with the coding sequence of the tetracycline repressor. To this end, the bGH poly-A-Ef1α-*GFP* fragment was amplified from DC-DON-SH01 using ATTCGACTCGAGTTCGAATTTAAATCGGATCCCT and ATTCGAGATATCGATCCGGTGGAGCCGGG and cloned into pBluescript SK (+) with *Xho*I/*Eco*RV to create pAN2067. Next, *GFP* was replaced by *TetR* amplified using PCR from pcDNA6/TR (Invitrogen) with ATTCGAAAGCTTGTGAGTTTGGGGACCCTTG and ATTCGAGATATCGCATAAGATCTGAATTCCCGGGA and inserted *Hin*dIII/*Eco*RV to generate pAN2070. Fragment bGH poly-A-Ef1α-*TetR* was released from pAN2070 with *Bst*BI/*Eco*RV and transferred to pAN2066 cut with *Bst*BI/*Nru*I to obtain pAN2071. Then, p97 or p97^QQ^ (gift from S. Fang) was amplified by PCR using GACTCGGATATCATGGCTTCTGGAGCCGATTCAA and TGTAACAACGTTTTAGCCATACAGGTCATCATCATCATT and cloned *Eco*RV/*Bst*BI into pAN2071.

#### Cell culture and generation of cell lines

SH-SY5Y cells were cultured in 5% CO_2_incubator at 37°C in high glucose DMEM (Sigma, D6546) containing 15% Tet System Approved FBS (Clontech, 631106), supplemented with 2 mM L-glutamine (Sigma, G5713), and 1 mM sodium pyruvate (Sigma, G7513). To generate stably transfected SH-SY5Y cells expressing p97 or p97^QQ^ under control of the Tet-On promoter, cells were transfected using Effectene (Qiagen, 301425) according to manufacturer's recommendations with two TALEN constructs for the PPP1R2C or AAVS1 “safe harbor” locus and an expression construct containing p97 or p97^QQ^ under control of the Tet-On promoter as well as a puromycin resistance gene to enable selection. For stably transfected cells, 0.75 μg/ml puromycin was added to maintain selection (Invivogen, ant-pr-1). Expression of p97 or p97^QQ^ was induced by treatment with 1 μg/ml tetracycline (Roth, Hp63.1). Mitochondrial stress was induced by treatment with 5 μM rotenone (Sigma, R8857), 75 μM 6-hydroxdopamine (Sigma, H8523) or 50 μM amyloid-β protein fragment 25–35 (Sigma, A4559). For pharmacological inhibition of p97 function, SH-SY5Y cells were treated with the p97-inhibitor DBeQ (Sigma, SML0031) at 2.5 μM for 20 h before further analysis (Chou et al., 2011).

### Flow cytometry

Flow cytometry was performed using a CyAn ADP Analyzer (Beckman Coulter). SH-SY5Y cells grown in 6 well cell culture plates (Sarstedt) and induced and/or treated as indicated were co-stained with DAPI and either 10 nM TMRE (Invitrogen) for 30 min at 37°C, or 5 μM MitoSOX™ (Invitrogen) for 10 min at 37°C. Cells were harvested, washed twice in PBS and resuspended in 1 ml PBS containing 0.5% (w/v) BSA and 50 μM EDTA. Flow cytometry was performed immediately afterwards. Data analysis was performed using FlowJo v.10. Sequential gating was performed as follows (with identical gates used for each experiment): cells were gated for using logarithmic forward/sideward scatter axes; doublet discrimination was performed using forward scatter area/forward scatter followed by pulse width/forward scatter dot plots. Dead cells were excluded in DAPI/forward scatter dot plots.

### Isolation of mitochondria

The human mitochondria isolation kit (Miltenyi Biotec, 130-094-532) was used according to manufacturer's instructions. Briefly, cells were harvested and either directly processed or stored overnight in liquid nitrogen. All following steps were performed on ice with pre-cooled buffers. Cells were resuspended in 800 μl lysis buffer supplemented with protease inhibitors (1 μg/ml pepstatin; 1 μg/ml leupeptin; 1 mM PMSF) and 50 μM EDTA. Cells were passed 15 times through a 25 gage needle. Nine milliliter separation buffer and 50 μl Anti-TOMM22 MicroBeads were added to the cell homogenate before rotating the suspensions for 1 h at 4°C. Magnetic separation was performed using a MACS Separator. Purified mitochondria were immediately lysed in RIPA buffer (Thermo Scientific) supplemented with protease inhibitors (1 μg/ml pepstatin; 1 μg/ml leupeptin; 1 mM PMSF) and 50 μM EDTA and subjected to five 10 s intervals of sonication at 10 kHz. Total protein content was measured using the Pierce BCA protein assay kit (Thermo Scientific) and was immediately followed by DNPH labeling.

### Labeling with DNPH

Labeling with DNPH was performed according to Wehr and Levine (2013) with minor alterations. Briefly, 60 μg of total protein in 20 μl total volume was added to 20 μl 12% sodium dodecyl sulfate (SDS). 40 μl of 20 mM DNPH in 2 M HCl were added, samples were briefly mixed and incubated for 15 min. To control for DNPH-reactive protein carbonyls, samples were reacted with 2 M HCl lacking DNPH. 30 μl of 2 M Tris base, 30% glycerol and a final concentration of 50 mM 1,4-Dithiothreit were added to stop the reaction. Samples were briefly mixed again and immediately used for SDS PAGE.

### Western blot

DNPH-labeled protein lysates were resolved by SDS PAGE (5 μg total protein per lane were loaded) and blotted onto nitrocellulose membrane (Whatman). Fast Green FCF (Sigma Aldrich, F7252) was used to evaluate total protein loaded per lane as follows: nitrocellulose membranes were stained for 10 min in Fast Green FCF staining solution (0.001% FCF, 30% methanol, 7% acetic acid), followed by 10 min in destaining solution (30% methanol, 7% acetic acid) and then 10 min in water. An infrared laser scanner (LiCor) was used for detection of FCF. Following Fast Green FCF, membranes were blocked for 1 h in 3% (w/v) Top Block (Lubio science, TB232010) in PBS-Tween 20 (0.05%) and immune-detection using polyclonal rabbit anti-DNP antibodies (D9656 Sigma, 1:2000) and polyclonal goat anti-rabbit Dylight 800 (35521 Pierce, 1:6000) was performed and detected using an infrared laser scanner (LiCor) to obtain quantitative measurement. Each experiment was performed three times independently and each measurement was performed in triplicates. Image analysis was performed using ImageJ (Schneider et al., 2012). For the analysis of mitochondrial fractions by western blotting mouse anti-p97 (10R-P104a Fitzgerald, 1:1000), mouse anti-cytochrome *c* (556433 Pharmingen, 1:2000), and rabbit anti-calnexin (ab75801 abcam, 1:2000) were used and detected using anti-mouse HRP or anti-rabbit HRP (Thermo Scientific) and ECL Plus chemiluminescent reagent (1896327 Thermo Scientific).

### Microcopy

Cells were seeded in 6-well plates onto glass slides at 1 × 10^4^ cells/well in 2 ml culture medium. Samples were fixed using methanol-free electron microscopy grade 4% paraformaldehyde in PBS for 15 min at RT, permeabilized for 15 min at RT using 0.15% Trixon X-100 in PBS and blocked for 1 h in 10% BSA (w/v) in PBS. To visualize mitochondria, samples were then incubated with mouse anti-cytochrome *c* antibody (BD Biosciences 556432, 1:1000) overnight at 4°C and Alexa546-conjugated anti-mouse antibodies (Invitrogen A11003, 1:500) for 1 h at RT. Nuclei were stained by incubation with DAPI (Invitrogen, D1306, 1:1000) for 5 min right after Alexa546 incubation. Samples were mounted in mounting medium (Vectashield, H1000) and observed using a confocal microscope (Zeiss LSM Meta710, 63 × / 1.4 objective). Mitochondria fragmentation and Parkin-to-mitochondria translocation were assessed visually and quantified as percentage of control. The extend of mitochondrial fragmentation was judged based on comparison to untreated control cells with mitochondrial networks scored as fragmented if most mitochondria in a cell did no longer exhibit an elongated phenotype. All experiments were performed independently in triplicates.

### Statistical analysis

Statistical significance of differences was assessed using pair-wise *t*-tests with the adjustment for multiple comparisons according to Holmes as implemented in R (R Core Team, 2013). Significance is indicated with n.s. *p* > 0.05, ^*^ for *p* < 0.05, ^**^ for *p* < 0.01, and ^***^ for *p* < 0.001.

## Author contributions

Lei Fang, Charles Hemion, Claudia C. Bippes performed the experiments. Albert Neutzner conceived the study. Albert Neutzner, Lei Fang planned the experiments. Josef Flammer provided intellectual input. Josef Flammer, Lei Fang, Charles Hemion, Claudia C. Bippes, Albert Neutzner wrote the manuscript.

### Conflict of interest statement

The authors declare that the research was conducted in the absence of any commercial or financial relationships that could be construed as a potential conflict of interest.
