# Supplementary material for: Mitochondrial function in neuronal cells depends on p97/VCP/Cdc48-mediated quality control
Source: Front Cell Neurosci. 2015 Feb 2;9:16. doi: 10.3389/fncel.2015.00016 (PMC4313695; doi:10.3389/fncel.2015.00016)
Supplement: Supplementary file 1 [file Presentation1.PDF]

Supplementary Material

Mitochondrial function in neuronal cells depends on p97/VCP/Cdc48-mediated quality control

Lei Fang[1], Charles Hemion[1], Ana C. Pinho Ferreira Bento[1], Claudia C. Bippes[1], Josef Flammer[2], Albert Neutzner[1,2]

[1]Ocular Pharmacology and Physiology, Department of Biomedicine, University Basel, Basel, Switzerland
[2] Department of Biomedicine, University Basel, Basel, Switzerland

*** Correspondence:** Albert Neutzner, Ocular Pharmacology and Physiology, Department of Biomedicine, University Basel, Hebelstrasse 20, Basel, 4031, Switzerland.
albert.neutzner@unibas.ch

1.1. Supplementary Figures

Supplementary Figure 1. Detection of carbonylated mitochondrial proteins. Representative western blots for the detection of carbonylated mitochondrial proteins. SH-SY5Y cells stably expressing p97 or dominant-negative p97QQ under control of the Tet-On promoter were induced with tetracycline for 2 hours or left uninduced and treated with (A) vehicle, (B) 5 µM rotenone, (C) 75 µM 6 OHDA, or (D) 50 µM Aβ for an additional 6 hours. Mitochondria were isolated using anti-TOMM22 magnetic beads resulting in highly purified mitochondria. Mitochondrial proteins were derivatized using DNPH and analyzed by western blotting. Total protein was determined using Fast Green FCF staining and quantified using an infrared laser-based scanner. Protein oxidation was determined using anti-DNP antibody-based detection of DNPH-derivatized carbonylated proteins and also quantified by using infrared laser scanning. Shown is one representative membrane (three technical and three biological replicates) stained using Fast Green FCF to visualize total protein loading (upper panel) followed by anti-DNP western blotting (lower panel). The bars mark the areas quantified by image analysis using ImageJ.

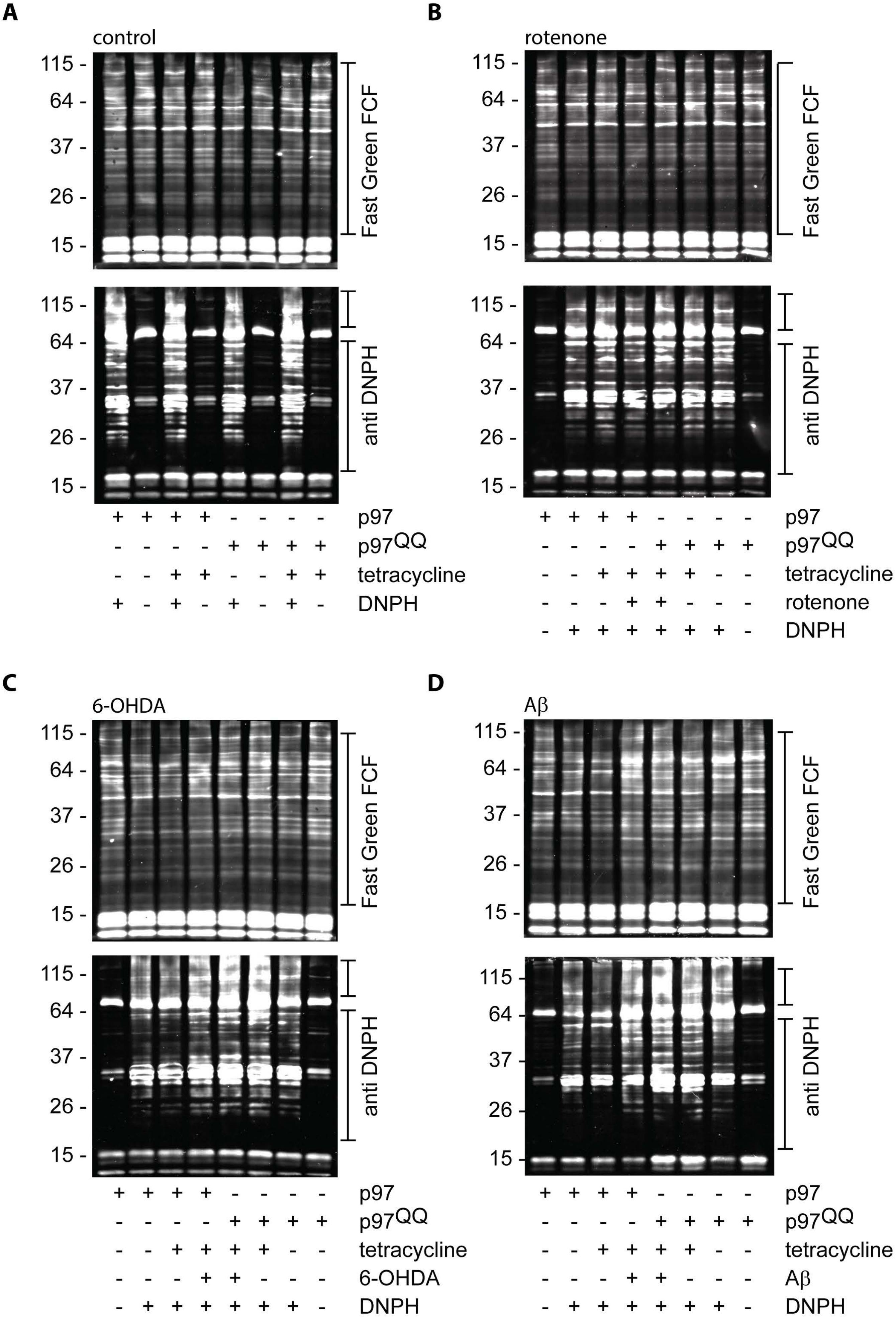

Figure S1 - Fang et al.
